# Healthy mitochondria attenuate metabolic dysfunction-associated steatohepatitis by restoring cell metabolism

**DOI:** 10.1186/s43556-025-00328-w

**Published:** 2025-10-11

**Authors:** Peiyu Zhou, Jingli Li, Yafang Xie, Xiaorong Li, Zhihong Cui, Ailing Fu

**Affiliations:** College of Pharmaceutical Sciences, Southwest University, Chongqing, China

**Keywords:** Metabolic dysfunction-associated steatohepatitis (MASH), Healthy mitochondria, Oxidative stress, Silent information regulator 1 (SIRT1)

## Abstract

Metabolic dysfunction-associated steatohepatitis (MASH) has become a major global health issue. Mitochondrial damage plays a crucial role in the development and progression of MASH. Therefore, it is speculated that mitochondrial transplantation therapy, which could replace dysfunctional mitochondria with normal ones, might potentially restore the liver cell metabolism of MASH. In palmitate-damaged AML-12 hepatocytes, exogenous mitochondria could eliminate lipid deposits and recover cell viability. However, in transforming growth factor β (TGF-β)-activated hepatic stellate cells (HSCs), the exogenous mitochondria showed the capability to inhibit the generation of α-smooth muscle actin (α-SMA) and collagen I. Moreover, the mechanism by which the exogenous mitochondria initiated the mitochondria-nucleus signaling pathway of liver cells was studied. The results showed the mitochondria could prevent metabolism disorders in the liver cells by regulating silent information regulator 1 (SIRT1) activity. Subsequently, a MASH animal model was established by the administration of a high-fat diet and the intraperitoneal injection of carbon tetrachloride to Kunming mice. The results indicated that the mitochondrial therapy significantly inhibited the livery injury and restored liver cell function in the experimental MASH mice (*p* < 0.01). The mitochondrial therapy would be a promising strategy to improve MASH pathological features, which could be developed as a new treatment option against MASH.

**Supplementary Information:**

The online version contains supplementary material available at 10.1186/s43556-025-00328-w.

## Introduction

Hepatocytes, which account for the vast majority of liver parenchymal cells, are the main victims of lipid metabolism disorders during metabolic dysfunction-associated steatohepatitis (MASH), whereas hepatic stellate cells (HSCs) are the key cells that drive liver fibrosis and disease progression [1–3]. Under normal circumstances, HSCs stand in a quiescent state. However, once they are activated, the cells could transform into myofibroblasts [4]. During the MASH stage, mitochondria exhibit structural and functional abnormalities in damaged hepatocytes [5, 6], including a reduction in oxidative phosphorylation (OXPHOS) and an increase in reactive oxygen species (ROS) levels [7–9]. Elevated ROS can impair the cell structure and induce the entry of HSCs from the quiescent state into the cell cycle [10, 11]. Therefore, a feasible way to prevent MASH would rescue hepatocytes from lipid injury and simultaneously inhibit the activation of HSCs.

However, the current treatment for MASH is not optimistic. The majority of candidate drugs, including selonsertib (an inhibitor of apoptosis signal-regulating kinase 1) and elafibranor (an agonist of peroxisome proliferator-activated receptors α/δ), have failed in clinical trials [12, 13]. Many drugs reported to improve mitochondrial function are ROS scavengers or metabolic regulators. Since the mitochondrial injury is present in hepatocytes during the MASH stage [14], ROS scavengers alone can only achieve limited effects. Similarly, metabolic regulators alone are hard to exert their desirable effects under the condition of mitochondrial injury.

Since mitochondrial dysfunction is closely related to MASH, supplementation of healthy mitochondria would be a feasible way to rescue the mitochondrial injury. The approach of mitochondrial transplantation therapy consists of transporting healthy mitochondria into hepatocytes, allowing healthy mitochondria to play a role in recovering cell function [15]. Compared with individual compounds, transported healthy mitochondria might exert multiple protective and repairing effects on cells. Mitochondrial transplantation therapy has developed rapidly in recent years and has been used to treat ischemic heart disease, Leigh syndrome, and neurodegenerative diseases [16–18]. The role of mitochondria has gone beyond just being a cell organelle to acting as a biological drug [19–21].

In this study, we suggested the mitochondrial transplantation therapy to prevent MASH progression. The effects of mitochondrial transplantation therapy were identified, and the underlying mechanism was revealed. The elucidation of the therapeutic effect and mechanism of healthy mitochondrial therapy on MASH could promote a new approach for the treatment of other metabolism-related diseases.

## Results

### Functional characterization of isolated mitochondria

**Fig. 1:**
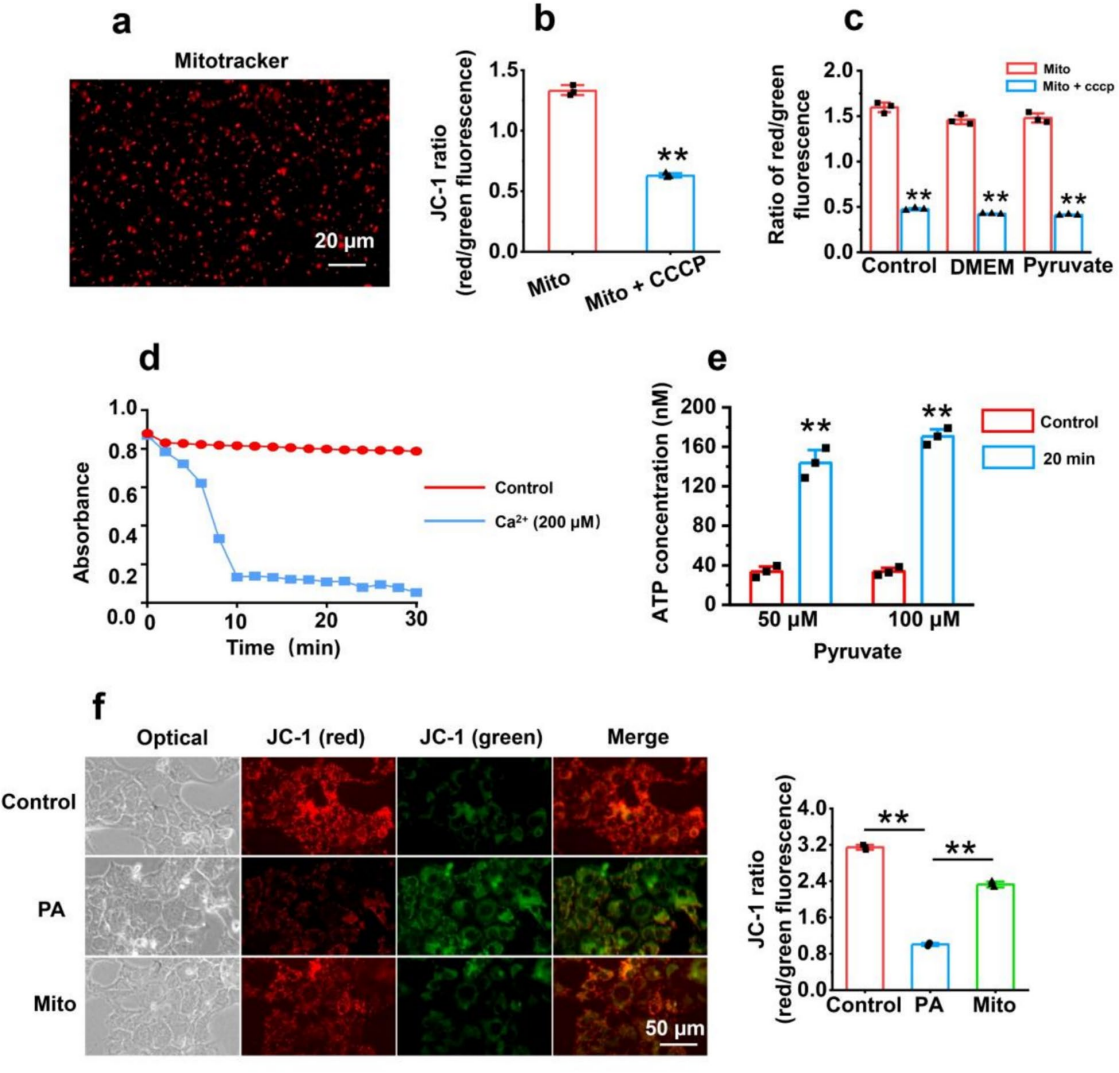
The activity of isolated mitochondria. **a** Mitochondria were labeled with MitoTracker Red CMXRos. **b** MMP was measured by JC-1. Mito, mitochondria; Mito + CCCP, mitochondria pretreated by CCCP. **c** MMP assay in DMEM and the medium supplemented with pyruvate. **d** Measurement of mitochondrial swelling. **e** Determination of ATP content. ATP content was measured at 0 (control) and 20 min after the mitochondria were incubated with the mixture containing glutamate, ADP, and phosphate ion. **f** The isolated mitochondria increased MMP in PA-damaged AML-12 hepatocyte cells. PA concentration, 200 μmol/L. PA, palmitate. Three independent experiments were conducted for each detection (*n* = 3). ** p < 0.01

Mitochondria were isolated from the livers of healthy mice using differential centrifugation. After separation, the mitochondria were stained with MitoTracker Red CMXRos and observed under a fluorescence microscope. The fluorescence image of the mitochondria revealed good dispersion (Fig. 1a). To verify the activity of the isolated mitochondria, the activity of the mitochondrial membrane potential (MMP; an indicator of mitochondrial function) was determined. The MMP was detected by JC-1 staining [22]. The results revealed that mitochondria had a significantly greater MMP than did carbonyl cyanide chlorophenylhydrazone (CCCP; an OXPHOS uncoupler)-treated mitochondria (*p* < 0.01) (Fig. 1b), no matter in mitochondrial isolation buffer, Dulbecco's modified eagle medium (DMEM), and the medium supplemented with pyruvate, evidenced by the ratio of red and green fluorescence intensity (Fig. 1c). In addition, the results of mitochondrial swelling detection suggested that the mitochondria appeared swollen and that the absorbance decreased after Ca^2+^ addition, whereas the control still had a relatively stable absorbance within 30 min (Fig. 1d). Moreover, the ability of mitochondria to produce ATP was determined by measuring ATP content. The results showed that the isolated mitochondria could generate ATP (Fig. 1e). These results indicated that the isolated mitochondria had an intact structure and activity.

To explore whether the mitochondria could enter liver cells and improve the intracellular MMP [23–25], JC-1 was used to stain the intracellular mitochondria. The images revealed that AML-12 hepatocytes damaged by palmitate (PA) presented relatively weak red fluorescence relative to that of the control, whereas the red fluorescence increased after the addition of the isolated mitochondria to the media (Fig. 1f). These results suggested that the mitochondria could increase the MMP in cells.

### Mitochondria alleviated lipid damage and rescued cell metabolism in hepatocytes

**Fig. 2:**
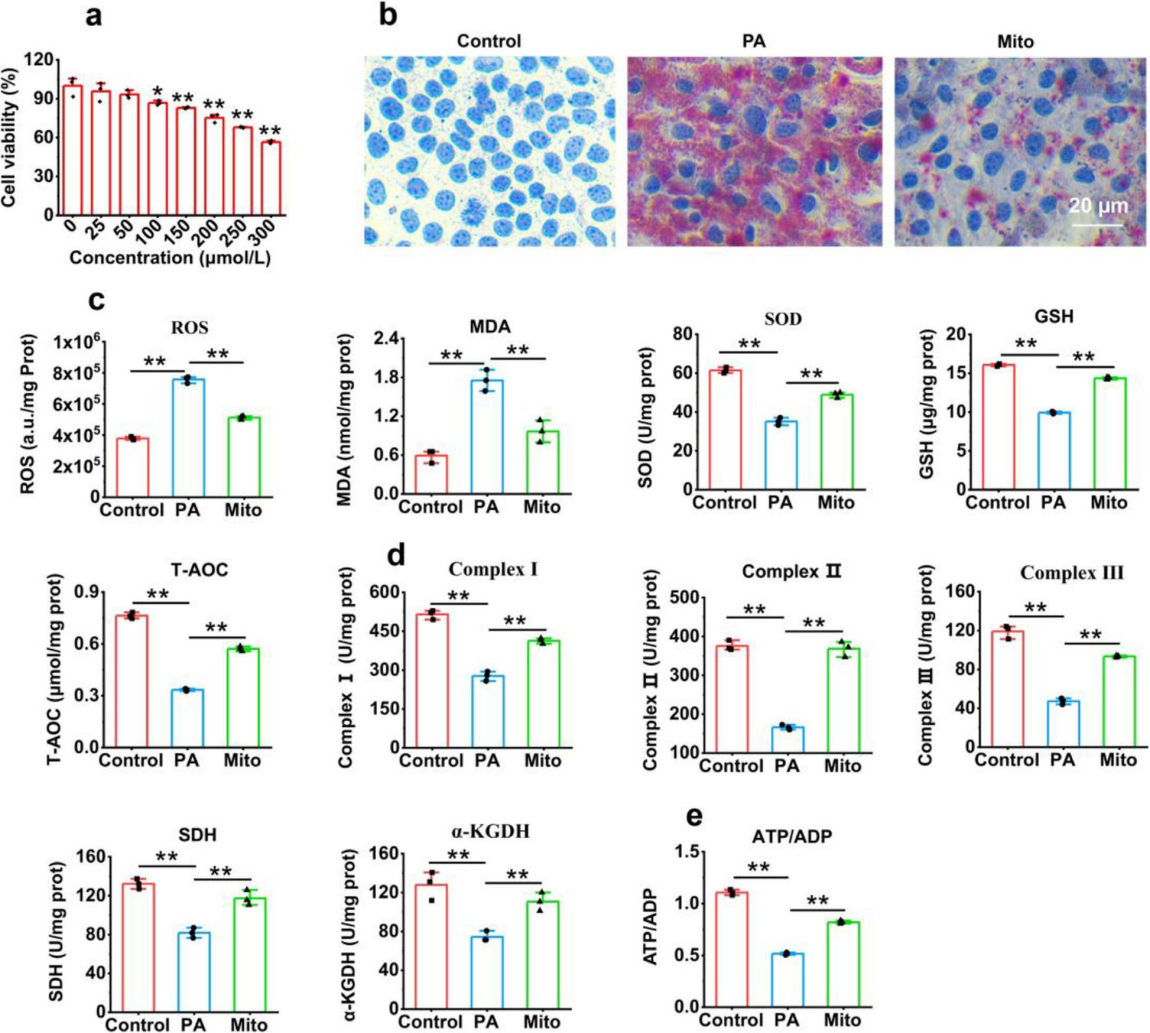
Exogenous mitochondria eliminated lipid deposition and alleviated oxidative damage in palmitate-damaged hepatocytes. **a** PA reduced the cell viability in a concentration-dependent manner. **b** Representative images of lipid accumulation in hepatocytes of each group after oil red O staining. PA concentration, 200 μmol/L. **c** Levels of ROS, MDA, SOD, GSH, and T-AOC. **d** Activities of mitochondrial complex I, II, and III, and SDH and α-KGDH in the TCA cycle. **e** ATP/ADP ratio. Three independent experiments were conducted for each detection (*n* = 3). * *p* < 0.05, ** *p* < 0.01

To examine the effect of isolated mitochondria on hepatocytes, a liver cell model was generated. When the AML-12 cells were incubated with PA, the cell viability decreased in a concentration-dependent manner, and the cell viability decreased to 70% as the PA concentration reached 250 μM (Fig. 2a). The images of oil red O staining revealed that a large amount of lipid deposition occurred in hepatocytes treated with PA, but after mitochondrial administration, lipid deposition was significantly reduced, with only scattered lipid particles distributed in the cells (Fig. 2b).

Since PA can induce oxidative stress to impair cell membranes and proteins [26], the levels of redox-related indices were tested to estimate the antioxidant activity of exogenous mitochondria. The results revealed that the levels of both ROS and malondialdehyde (MDA; the final product of lipid metabolism) decreased significantly after mitochondrial treatment (*p* < 0.01), whereas the levels of antioxidant components within cells, such as superoxide dismutase (SOD), glutathione (GSH), and total antioxidant capacity (T-AOC), increased (*p* < 0.01) (Fig. 2c), which suggested that the mitochondria could inhibit PA-induced oxidative damage. Moreover, the mitochondrial therapy increased the levels of intracellular mitochondrial complexes I, II, and III and the activities of succinate dehydrogenase (SDH) and α-ketoglutarate dehydrogenase (α-KGDH) (*p* < 0.01) (Fig. 2d). Correspondingly, adenosine triphosphate (ATP) production increased (*p* < 0.01) (Fig. 2e).

### Mechanism by which exogenous mitochondria recovered hepatocyte activity

**Fig. 3:**
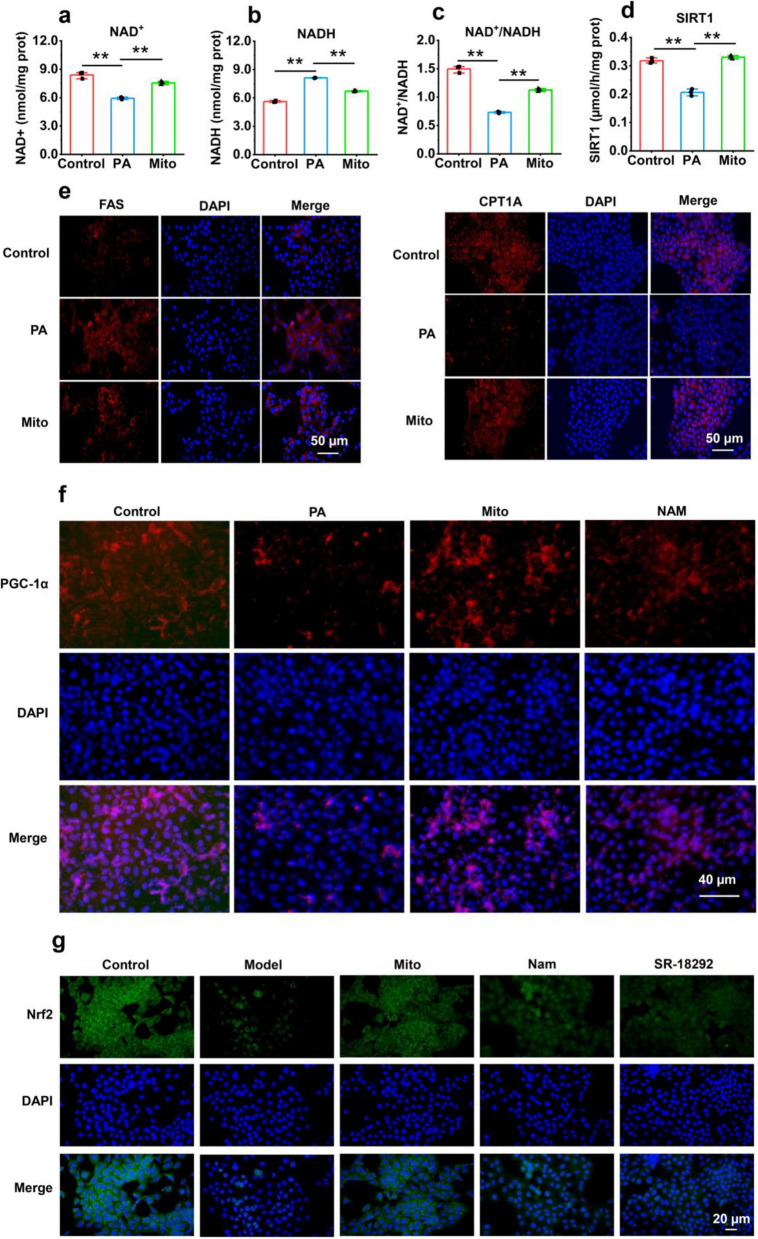
Exogenous mitochondria increased SIRT1 activity. **a** NAD^+^ level. **b** NADH content. **c** NAD^+^/NADH ratio. **d** SIRT1 activity. Three independent experiments were conducted for each detection (*n* = 3). * *p* < 0.05, ** *p* < 0.01. **e** The enzymes in lipid metabolism, FAS and CPT1A, were respectively detected by immunofluorescence staining. **f** Detection of PGC-1α by immunofluorescence staining. **g** Detection of Nrf2 by immunofluorescence staining

To elucidate the mechanism underlying mitochondrial therapy, nicotinamide adenosine dinucleotide (NAD^+^) and its regulated signals were examined. Mitochondria can convert reduced nicotinamide adenosine dinucleotide (NADH) to NAD^+^ through the respiratory chain complex I. After mitochondrial addition, the intracellular NAD^+^ content increased (*p* < 0.01), but the NADH level decreased according to high-performance liquid chromatography (HPLC) analysis (Fig. 3a and 3b), and the corresponding NAD^+^/NADH ratio increased (Fig. 3c). NAD^+^ is an essential ligand of NAD^+^-dependent silent information regulator 1 (SIRT1), a signaling protein that can regulate oxidative stress, autophagy, and inflammation to protect and repair cells [27]. The results revealed that SIRT1 activity increased after intracellular NAD^+^ content increased (*p* < 0.01) (Fig. 3d).

Moreover, the levels of fatty acid synthase (FAS; one of the main enzymes involved in fatty acid synthesis) and carnitine palmitoyl transferase 1 A (CPT1A; an enzyme in fatty acid decomposition) were determined by immunofluorescence staining. The images revealed that the FAS level increased in PA-damaged AML-12 cells, whereas the fluorescence intensity decreased after the addition of mitochondria (Fig. 3e). Additionally, the fluorescence intensity of CPT1A in AML-12 cells decreased after PA treatment but recovered after mitochondrial addition (Fig. 3e). These results suggested that healthy mitochondria could promote fatty acid decomposition and reduce lipid production in PA-damaged liver cells.

Peroxisome proliferator-activated receptor γ coactivator factor 1 (PGC-1) is a downstream target protein of SIRT1, and its α type (PGC-1α) is closely related to metabolism in liver [28, 29]. Thus, the level of PGC-1α was examined by Western blot (WB) and immunofluorescence staining. The results revealed that the PGC-1α level increased in mitochondria-treated cells, but nicotinamide (NAM; a SIRT1 inhibitor) and SR-18292 (a PGC-1α inhibitor) reversed the increase in the protein level (Fig. 3f, Fig. S2 and Fig. S3), indicating that the exogenous mitochondria would enhance the PGC-1α content through increasing SIRT1 activity. Since SIRT1 could also regulate the expression of nuclear factor-erythroid 2 related factor 2 (Nrf2) [30], the level of Nrf2 was measured by immunofluorescence staining and WB. As expected, the content of Nrf2 increased in the mitochondria-treated cells, but NAM and SR-18292 reduced the Nrf2 levels in the cells (Fig. 3g and Fig. S4).

### Exogenous mitochondria inhibited the production of fibrosis protein in HSCs

**Fig. 4:**
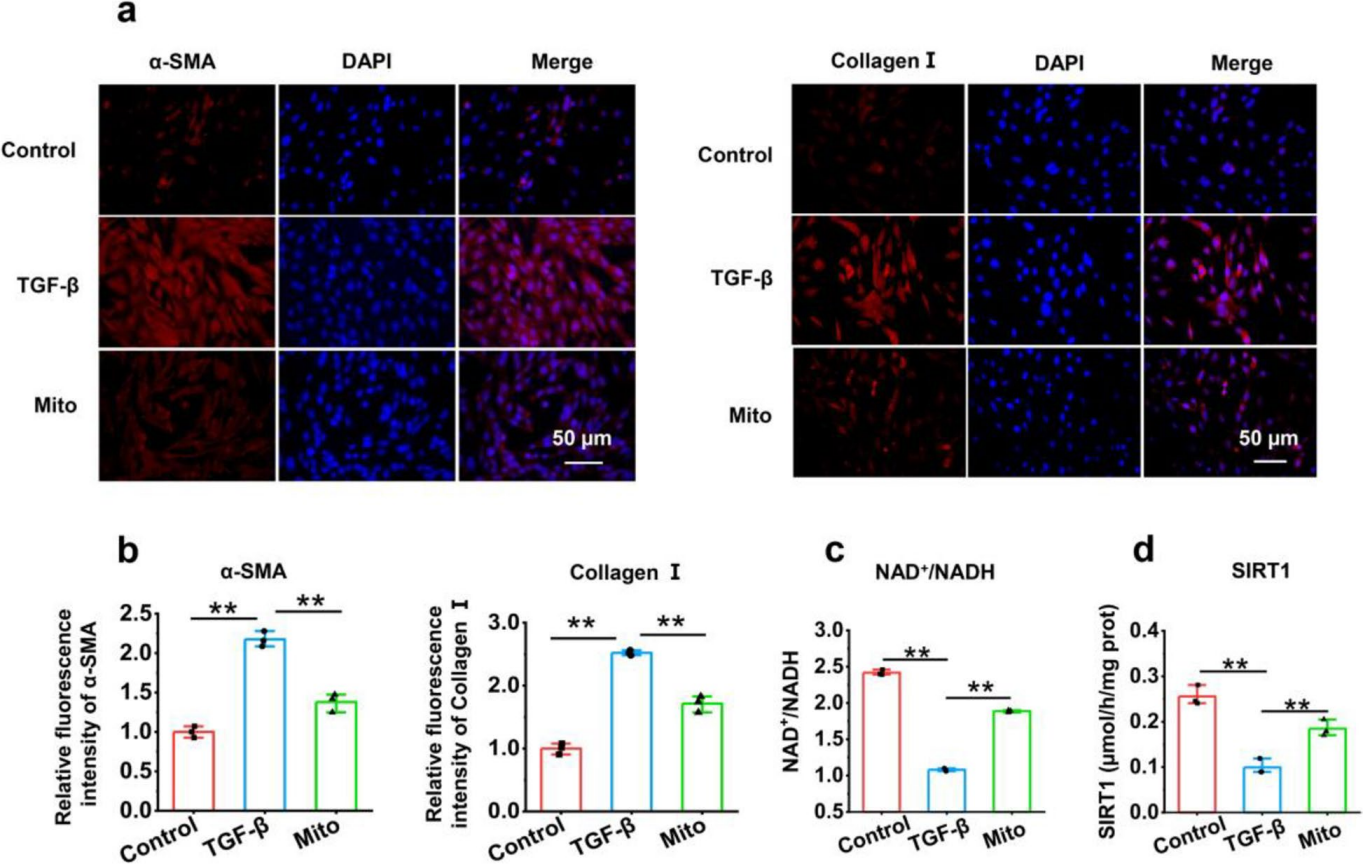
Exogenous mitochondria inhibited the production of α-SMA and collagen I in HSCs. **a** Liver fibrosis indicators, α-SMA and collagen I, were respectively assayed by immunofluorescence staining. **b** The average fluorescence intensity of α-SMA and collagen I. The average fluorescence intensity values were calculated using Image J. **c** NAD^+^/NADH ratio. **d** SIRT1 activity. Three independent experiments were conducted for each detection (*n* = 3). * *p* < 0.05, ** *p* < 0.01

To investigate the effect of mitochondrial therapy on HSC activation, transforming growth factor β (TGF-β)-activated cells were used. After mitochondrial addition, the marker proteins in liver fibrosis, α-smooth muscle actin (α-SMA) and collagen I, were determined by immunofluorescence staining. The images revealed that the fluorescence became strong after TGF-β promotion but significantly decreased after mitochondrial addition (Fig. 4a and 4b), suggesting that mitochondria prevent the expression of liver fibrosis proteins in activated HSCs.

To identify whether NAD^+^/SIRT1 would be involved in restoring cellular function, the concentrations of intracellular NAD^+^ and NADH were detected by HPLC. The results revealed that the NAD^+^ level increased and the NADH level decreased after mitochondrial administration, increasing the ratio of NAD^+^/NADH (Fig. 4c). Moreover, SIRT1 activity in mitochondria-treated cells was markedly greater than that in TGF-β-activated cells (*p* < 0.01) (Fig. 4d).

### Healthy mitochondria improved lipid metabolism in experimental mice with MASH

**Fig. 5:**
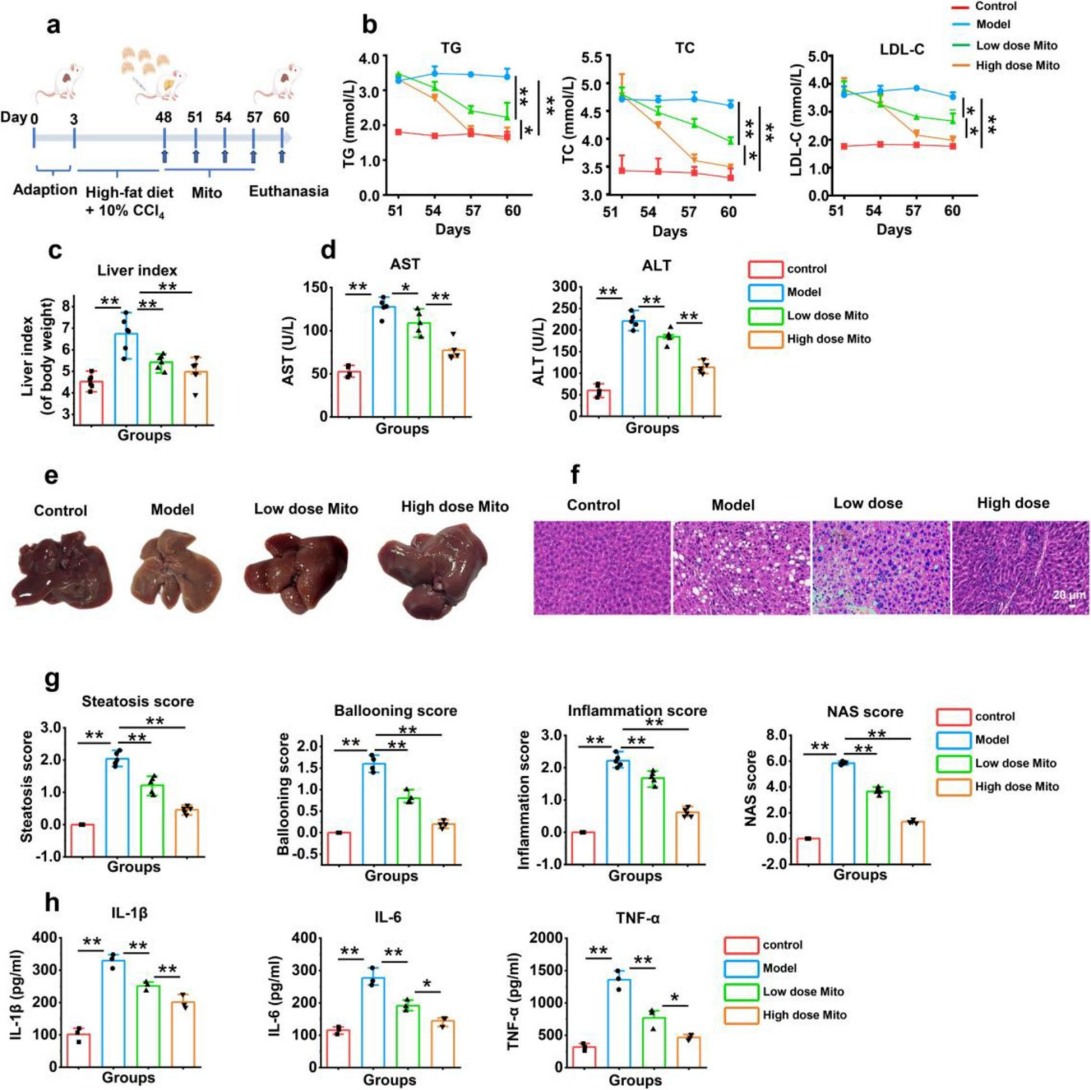
Exogenous mitochondria alleviated liver damage in the MASH model mice. **a** Schedule of mitotherapy for the MASH mice. **b** Levels of TG, TC, and LDL-C in serum. **c** Liver index of mice in each group. **d** Levels of AST, ALT in mouse sera. **e** Representative liver images of each group of mice. **f** Representative HE staining of liver tissue. **g** Scoring of steatosis, balloon-like transformation, lobular inflammation, and NAS in liver tissues. **h** Contents of inflammatory cytokines, IL-1, IL-6, and TNF-α in serum. * *p* < 0.05, ** *p* < 0.01

To determine the in vivo distribution of exogenous mitochondria after intravenous injection, the mitochondria were labeled with MitoTracker Red CMXRos. The results showed that mitochondria could distribute in animal tissues, including liver (Fig. S1). To examine whether healthy mitochondria could improve the liver function of animals with MASH, an experimental MASH model was generated with a high-fat diet and intraperitoneal injection of carbon tetrachloride (CCl_4_). After 45 days of preparation of this mouse model preparation, healthy mitochondria with low-dose (5 mg/kg body weight) and high-dose (10 mg/kg body weight) mitochondria were administered to the model mice every three days for a total of 12 days, during which mitochondria were injected 4 times in total (Fig. 5a). Compared with those in the model group, the levels of TG, total cholesterol (TC), and low-density lipoprotein cholesterol (LDL-C) in the serum of the mice treated with mitochondria were significantly lower (Fig. 5b). Mitochondrial therapy also improved the liver index (an indicator for evaluating liver injury) (Fig. 5c). Additionally, the mitochondria significantly reduced the levels of aspartate aminotransferase (AST) and alanine aminotransferase (ALT) in the sera of mice with MASH (*p* < 0.01) (Fig. 5d).

After the liver tissues in each group were separated, the normal liver appeared reddish brown in color, whereas the livers of the mice with MASH appeared pathological abnormality (Fig. 5e), exhibiting typical MASH lesions. The liver tissue was subsequently sectioned and stained with hematoxylin–eosin (HE), and the images revealed that the liver lobular structure of the MASH model mice was disordered (Fig. 5f). However, liver appearance and the liver index recovered after mitochondrial therapy. The pathological scoring of digital images revealed that the mitochondria reduced steatosis, balloon-like transformation, and lobular inflammation in the liver tissues of MASH model mice (Fig. 5g).

Moreover, the concentrations of the serum inflammatory cytokines IL-1β, IL-6, and TNF-α in the mice in the mitochondria treatment group were lower than those in the model mice (*p* < 0.01) (Fig. 5h). These results suggested that healthy mitochondria could inhibit inflammatory cytokines and liver fibrosis in MASH model mice.

### Healthy mitochondria inhibited the fibrosis of liver tissues in MASH model mice

**Fig. 6:**
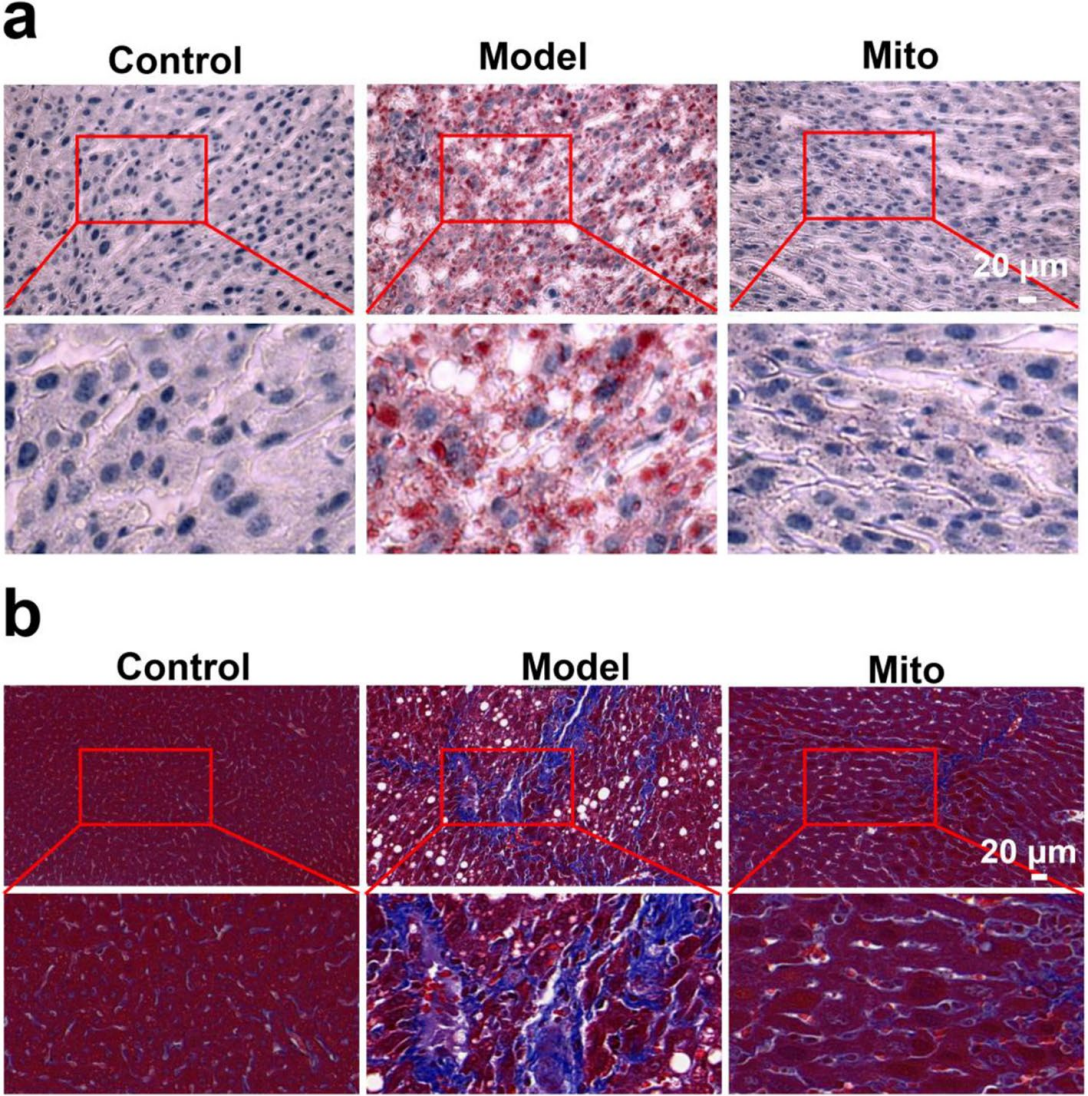
Healthy mitochondria suppressed liver fibrosis in the MASH model mice. Representative pictures of (**a**) oil red O and (**b**) Masson staining of liver tissues

To determine the effects of healthy mitochondria on fat and collagen fiber contents in the liver, oil red O and Masson staining were used. The oil red O images revealed that the livers of the model mice contained large amounts of lipid droplets (Fig. 6a), but fat accumulation decreased after mitochondrial administration (10 mg/kg body weight). Similarly, healthy mitochondria reduced the content of collagen fibers in the liver tissue of mice with MASH (Fig. 6b). These results were consistent with those of Masson’s trichrome staining, indicating that mitochondria can reduce collagen production in animals with MASH.

### Healthy mitochondria restored cell metabolism

**Fig. 7:**
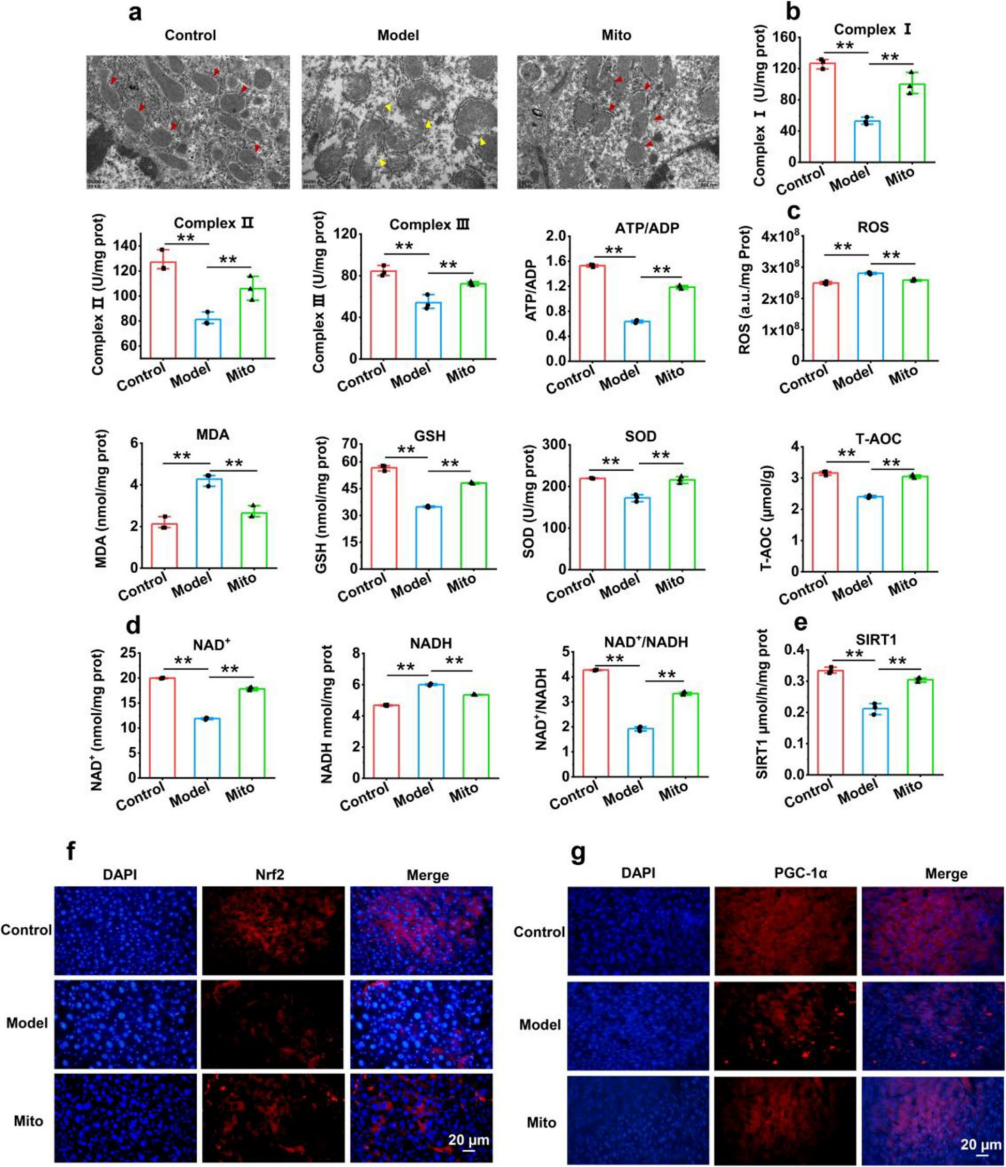
Mitochondrial therapy restored the liver cell metabolism. **a** Liver mitochondria were observed under TEM. **b** Levels of mitochondrial complex I, II, and III, as well as ATP/ADP, were respectively tested in the mouse liver of each group. **c** Levels of oxidative stress indicators, ROS, MDA, GSH, SOD, and T-AOC, were determined in the mouse liver respectively. **d** Contents of NAD^+^ and NADH, as well as the ratio of NAD^+^/NADH, were detected in each group. **e** SIRT1 activity. **f** Nrf2 and **g** PGC-1α were respectively assayed by immunofluorescence staining. * *p* < 0.05, ** *p* < 0.01

A transmission electron microscope (TEM) was used to observe the mitochondrial morphology of the liver. TEM revealed that the liver mitochondria in the control and mitochondria-treated groups were intact, whereas the livers of mice with MASH showed vacuoles and swelling (Fig. 7a). The function of cell metabolism was estimated in the livers of MASH model mice by the activities of complexes I, II, and III, as well as the ratio of ATP and adenosine diphosphate (ADP). The results revealed that the levels of the complexes and ATP/ADP were significantly increased in the livers of mitochondria-treated mice (*p* < 0.01) (Fig. 7b), suggesting that mitochondrial function was improved after healthy mitochondrial treatment. Moreover, the contents of liver ROS and MDA decreased, whereas the GSH level and SOD activity increased, resulting in an increase in the T-AOC level of the cells after mitochondrial administration (Fig. 7c).

To verify that the SIRT1/PGC-1α signaling pathway participated in mitochondrial treatment, the contents of NAD^+^ and NADH were examined. The results indicated that NAD^+^/NADH was elevated in the livers of mitochondria-treated mice (Fig. 7d), and correspondingly, SIRT1 activity significantly increased (Fig. 7e), which was consistent with the results of the in vitro study. Additionally, immunofluorescence staining and WB revealed that Nrf2 and PGC-1α levels were increased in the livers of MASH model mice after the mice received mitochondrial treatment (Fig. 7f, Fig. 7g, and Fig. S5).

## Discussion

**Fig. 8:**
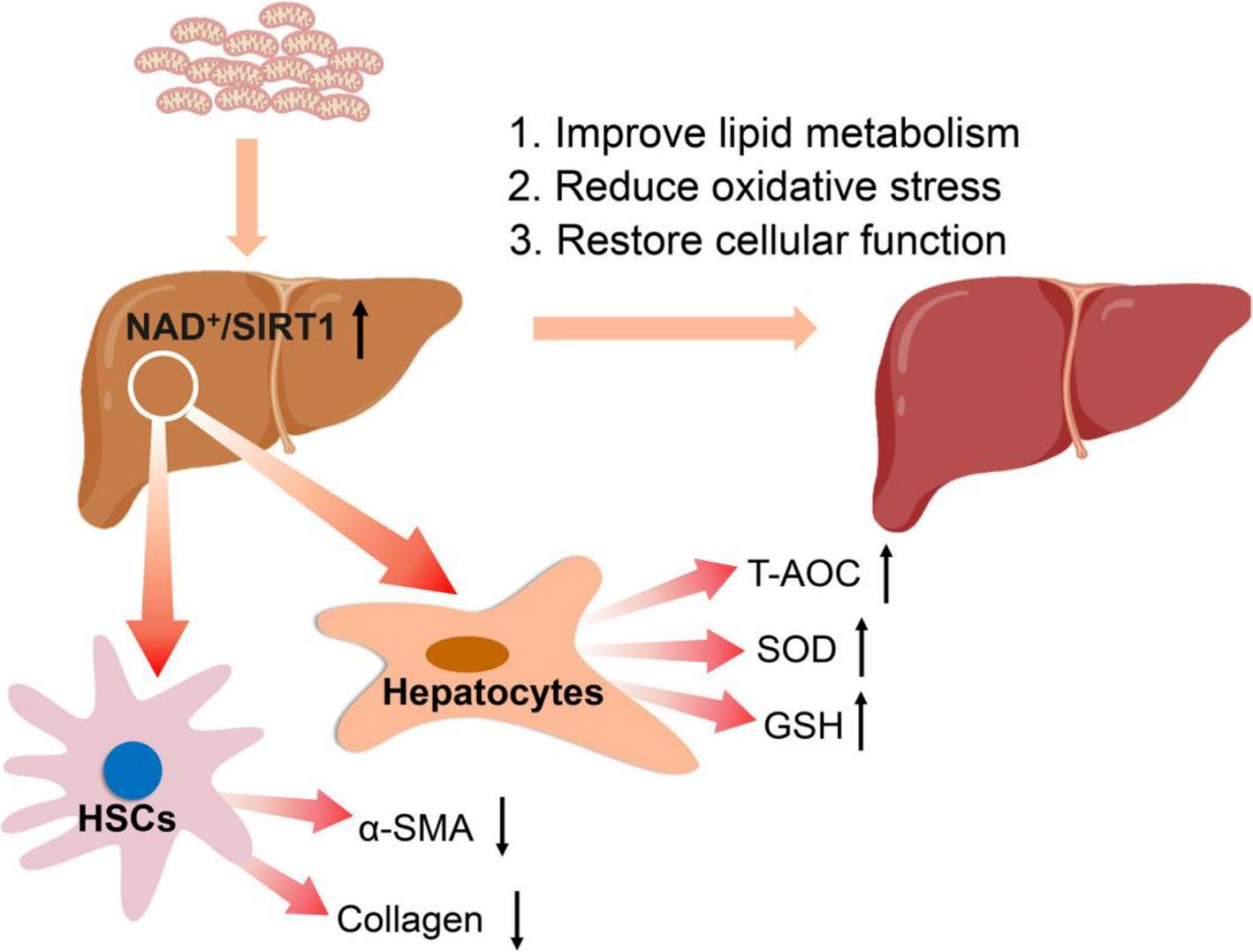
Exogenous mitochondria could improve liver cell metabolism by regulating SIRT1 activity. The picture was drawn by using Generic Diagramming Platform (GDP, https://BioGDP.com) [52]

MASH has become the leading chronic liver disease worldwide. Despite numerous attempts to prevent disease progression, there is still a lack of effective therapeutic agents. Since mitochondria play a crucial role in hepatocyte injury, restoring mitochondrial function in cells is possible. In this study, healthy mitochondria were transported into impaired hepatocytes, and the rescue effect of healthy mitochondria on the recovery of cell metabolism through regulating SIRT1 activity (Fig. 8). Additionally, the potential therapeutic effect of healthy mitochondria was achieved in experimental animals with MASH. Healthy mitochondria could exert their effects through two main mechanisms: one is to improve the ATP supply and cellular metabolic capacity, and the other is to regulate the expression of genes by signal pathways. This study provides a feasible approach for treating MASH and other liver metabolism-related diseases.

Healthy mitochondrial transplantation therapy has become an emerging approach for disease treatment in recent years, whose main purpose is to replace damaged mitochondria in cells with healthy mitochondria to rescue cell function [31]. This novel therapeutic method has developed rapidly [32]. Current reports indicate that mitochondria not only repair damaged normal cells, but also control inflammatory factor release and tumor cell proliferation [33, 34].

During MASH, liver cells exhibit significant lipid deposition accompanied by ROS production [35, 36]. Free fatty acids could be among the raw materials for healthy mitochondria and decompose fatty acids to supply energy. In this study, the fatty acid content significantly decreased after mitochondrial supplementation, as did the levels of ROS and MDA. Moreover, mitochondria could serve as the center of signal transduction, releasing signal molecules to the nucleus and regulating the expression of nuclear genes [5]. This mitochondria-nuclear interaction plays a profound role in maintaining normal cell activity. However, the interaction is decreased due to mitochondrial injury in the liver cells of MASH.

To elucidate whether supplemented mitochondria play signal hub roles in liver cells, NAD^+^, an important molecule in mitochondrial metabolism, was considered in this study. NAD^+^ is a mitochondrial metabolite that is converted from NADH by the dehydrogenase of the mitochondrial respiratory chain. NAD^+^ can function as a specific ligand for SIRT. Then, NAD^+^/SIRT can be transferred from the cytoplasm to the nucleus, where it regulates the acetylation level of target proteins. The SIRT family comprises 7 subtypes, in which SIRT1 is relatively abundant in the liver. Liver-specific SIRT1 knockout mice can develop liver diseases such as hepatic steatosis, hepatitis, and MASH [37, 38]. Reports also show that NAD^+^ reduction could result in a decrease of SIRT1 activity [39–41]. In activated HSCs, glycolysis increases, and NAD^+^ decreases, which could cause a decrease in the NAD^+^/NADH ratio, leading to a decrease in SIRT1 activity [42, 43]. In this study, we revealed that healthy mitochondria could increase NAD^+^ production, accompanied by increased SIRT1 activity.

Both the inhibition of HSC activation and the induction of HSC apoptosis can alleviate liver fibrosis, but the signaling pathways involved may vary. The TAK1/MAPK/FoxO3a pathway is reportedly involved in inducing apoptosis in HSCs [44], whereas the SIRT1-mediated pathway may play an important role in inhibiting cell activation [38, 45]. PGC-1α is one of the target proteins of NAD^+^/SIRT1 in liver metabolism, whose main function is to protect and repair cells, such as inhibiting oxidative stress, and inflammatory response, and maintaining mitochondrial homeostasis [46]. The PGC-1α signal could also induce transcription of autophagy-related genes and stimulate the expression of Pink1/Parkin [47]. Therefore, healthy mitochondria inhibit the progression of MASH through the dual effects: in terms of metabolism, mitochondria decompose lipids in liver cells to reduce lipid accumulation; In terms of signal transduction, mitochondria increased SIRT1 activity to restore cell activity.

In summary, MASH is an increasingly serious global health problem, which is a accelerating toward more severe disease progression. However, there are almost no specific drugs for treating MASH. Thus, the development of a new and effective strategy to treat this disease is urgently needed. Owing to the complexity of the pathogenesis by which both hepatocyte damage and HSC activation are involved in the development of MASH, it is difficult to develop drugs that target a particular type of cell. Although hepatocytes and HSCs exhibit different directions during disease progression, both show metabolic abnormalities in MASH. In the present study, healthy mitochondria improved cell metabolism through increasing SIRT1 activity. The study does not exclude other mechanisms involved in mitochondrial therapy. The limitations of the study include the pathways through which cells uptake mitochondria, as well as the interactions between mitochondria and other organelles within cells, which need to be further clarified. Nevertheless, this study not only suggests that healthy mitochondrial therapy is a potential way to treat MASH, but also indicates that aiming at both hepatocytes and HSCs simultaneously could be a feasible approach to block the progression of MASH.

## Materials and Methods

### Animals

Healthy male Kunming mice (4 - 5 weeks old), weighing 20 - 24 g, were obtained from the Animal Experiment Center of Chongqing Medical University. The mice were raised in a specific pathogen-free (SPF) animal laboratory (22 - 25 °C) with free access to drinking water. The mice were housed in standard culture cages with 5 mice *per* cage. The animal study was approved by the Animal Care and Use Committee of Southwest University ((IACUC-20230925-01).

### Separation and staining of mitochondria

Liver mitochondria from healthy mice were isolated through differential centrifugation at 4 °C according to previous methods [48]. Briefly, the liver tissue was rapidly separated after the mice were euthanized. The tissues were homogenized in SE buffer (0.25 M sucrose, 10 mM EDTA-2Na, 30 mM Tris, 2.5 mM CaCl_2_). The homogenization mixture was centrifuged at 3000 rpm twice to remove the precipitate. The supernatant was subsequently centrifuged twice at 12,000 rpm for 15 min each time. The precipitate was collected and suspended in phosphate buffer to be stained with MitoTracker Red CMXRos (Beyotime Biotechnology, China), and a fluorescence microscopy (Chongqing Optec Instrument Co., Ltd., China) was used to observe the mitochondrial fluorescence. Mitochondrial swelling was detected by using CaCl_2._ The ability of the isolated mitochondria to generate ATP was assayed as previously described [16].

### Measurement of the MMP

The MMP, an index that reflects the integrity of isolated mitochondrial membranes, was tested by JC-1 staining. JC-1 is a fluorescent dye that can enter healthy mitochondria to form aggregates with red fluorescence, whereas JC-1 exhibits green fluorescence as a monomer as the MMP decreases. After the mitochondria were incubated with JC-1 at 37 °C for 15 min, the fluorescence intensity was tested by a fluorescence spectrometer (Hitachi, Japan). The mitochondria treated with CCCP were used as the control.

### Cell culture

AML-12 hepatocytes stored in the laboratory were identified by the short tandem repeat‌ authentication. AML-12 cells grew in the DMEM/F12 media with 10% fetal bovine serum (FBS), 1% penicillin/streptomycin, 1% insulin-transferrin-selenium, and 40 ng/ml dexamethasone. JS-1 hepatic stellate cells were cultured in DMEM high glucose media with 10% FBS [49]. The cells were placed in a cell culture incubator (Esco Life Sciences, Singapore) supplemented with 5% CO_2_ at 37 °C. All materials for cell culture were purchased from Wuhan Pricella Biotechnology. Co., Ltd. (Wuhan, China).

### Palmitate-induced hepatocyte injury

AML-12 cells (1 × 10^5^) in the logarithmic growth stage were incubated in 96-well plates. When the cells reached 70–80% confluency, a PA solution at 0, 25, 50, 100, 150, 200, 250, or 300 μmol/L concentration (dissolution method described in the supplementary materials) was added to the media to induce lipid damage in the hepatocytes. After 24 h of incubation, the cell viability was assayed with 3-(4,5-dimethylthiazol-2-yl)−2,5-diphenyltetrazolium bromide (MTT). The absorbance (OD) was subsequently measured at 560 nm using a microplate reader (Thermo Fisher Scientific, USA). The cells treated with phosphate buffer solution (PBS) were used as controls. Cell viability was calculated according to the following formula: viability (%) = (OD_sample_/OD_control_) × 100%.

### Biochemical measurement

The levels of redox-related indicators, including ROS, T-AOC, SOD, MDA, and GSH, were measured individually by commercial kits (Beijing Solarbio Science & Technology Co., Ltd, China). Briefly, the ROS concentration was examined by DCFH-DA, and the T-AOC was detected via ferric reducing ability determination, SOD activity with nitrogen blue tetrazole photochemical reduction, the GSH content with 5,5'-dithiobis-2-nitrobenzoic acid, and the MDA level using a thiobarbituric acid-reactive substance. The enzyme activities of the mitochondrial TCA cycle, α-KGDH, and SDH, as well as those of complexes I, II, and III, respectively, were examined on the basis of the protocol of the operating manuals (Beijing Solarbio Science & Technology Co., Ltd, China). The contents of NAD^+^, NADH, ATP, and ADP were quantified by HPLC. SIRT1 activity detection kit was purchased from Shanghai Jiemei Gene Pharmaceutical Technology Co., Ltd, China (catalog number GMS 50287.1). Moreover, the levels of TG, TC, and LDL-C, as well as the activities of AST and ALT, were detected on an automatic biochemical analyzer. The levels of the serum inflammatory cytokines IL-1β, IL-6, and TNF-α were respectively determined using enzyme-linked immunosorbent assay kits [50] (‌Shanghai Hengyuan Biotechnology Co., Ltd., China).

### Oil red O staining

AML-12 cells were washed with PBS (pH 7.5) twice and then fixed with formaldehyde for 20 min. After being washed sequentially with distilled water and 60% isopropanol, the cells were stained with 0.5% Oil red O for 15 min. Subsequently, the staining solution was discarded, and the cells were rinsed with 60% isopropanol or distilled water. After the nuclei were stained with Mayer’s hematoxylin solution, the cells were observed under the microscope (Chongqing Optec Instrument Co., Ltd., China).

### Immunofluorescence

HSCs and AML-12 cells were incubated in 24-well plates. When the cells grew to 70 - 80% confluency, TGF-β solution (5 ng/mL) or PA solution (200 μmol/L) was added to the cell culture medium to induce HSC activation or AML-12 damage, respectively. After treatment for 24 h, mitochondria (50 μg) were added to the media for further incubation for 6 h. The cells were fixed with methanol for 10 min and then rinsed with PBS 3 times. After the cells were incubated with 5% bovine serum albumin at room temperature to block nonspecific proteins, primary antibodies against α-SMA (1:300), collagen I (1:800), FAS (1:1000), CPT1A (1:600), Nrf2 (1:200), and PGC-1(1:2000) were added to the blocking buffer for incubation overnight. The cells were subsequently washed with PBS, and the FITC-labeled goat anti-rabbit IgG secondary antibody was added to the incubation buffer for 2 h in the dark. After the cell nuclei were labeled with 4′,6-diamino-2-phenylindole (DAPI), the cells were observed under a microscope. Fluorescence images were taken with the fluorescence microscope.

### Generation of experimental MASH model mice and mitochondrial therapy

The mice were randomly divided into four groups: the control group, model group, low-dose mitochondrial group (5 mg/kg body weight), and high-dose mitochondrial group (10 mg/kg body weight). Six mice were included in each group, with 24 mice in total. The animal model was prepared with a high-fat diet (feed ingredients and catalog number in supplementary materials) combined with CCl_4_, in which 10% CCl_4_ in olive oil was injected intraperitoneally every three days [51]. The control mice were given a normal diet and an equal volume of olive oil. After 45 days of administration, the mice in the low-dose mitochondrial group were injected intravenously with mitochondria (5 mg/kg body weight), and the animals in the high-dose mitochondrial group were given 10 mg/kg mitochondria. During this period, blood samples were taken every three days for the measurement of plasma TG, TC, and LDL-C contents. After mitochondrial administration once every three days for a total of 15 days, the mice were euthanized, and the liver was dissected for weighing, histological identification, and biochemical determination. The liver index (liver relative weight) was calculated as the liver weight/mouse body weight ratio. The liver tissues were stained with HE and Masson’s trichrome, respectively. For Masson staining, the paraffin sections were deparaffinized sequentially with xylene, anhydrous ethanol, 90% ethanol, 80% ethanol, 70% ethanol, and distilled water. Then, the slices were immersed in Bouin solution overnight to increase tissue permeability. After being washed with water, the slices were stained with turquoise blue, hematoxylin, or phosphomolybdic acid solution for 3 min. Then, aniline blue staining solution was added to the slices for 30 s to further optimize the color of the tissue. Afterward, the slices were dehydrated with 95% ethanol or anhydrous ethanol and cleared in xylene for 2 min. The slices were sealed and then observed under a microscope.

The images were analyzed by using a Pannoramic MIDI II digital slice scanner (Jinan Danjier Electronics Co., Ltd., China) to obtain digital images, and then pathological changes were scored as steatosis (0 - 3 points), hepatocyte ballooning (0 - 2 points), and lobular inflammation (0 - 3 points) according to the NAS scoring. In addition, pieces of liver tissue from each group were individually immersed in 2.5% glutaraldehyde for TEM observation and photography.

### Statistical analysis

The data are expressed as the means ± SDs and were analyzed using SPSS and Origin. T tests and one-way ANOVA were used to compare the differences between groups. Significant and highly significant differences are indicated as * *p* < 0.05 and ** *p* < 0.01, respectively.

## Supplementary Information

Supplementary Material 1.

## Data Availability

All the data supporting the findings of this current study are available from the corresponding author upon reasonable request.

## Abbreviations

ADP: Adenosine diphosphate
ATP: Adenosine triphosphate
ALT: Alanine aminotransferase
AST: Aspartate aminotransferase
BSA: Bovine serum albumin
CCCP: Carbonyl cyanide chlorophenylhydrazone
CCl_4_: Carbon tetrachloride
DMEM: Dulbecco's modified eagle medium
FBS: Fetal bovine serum
GSH: Glutathione
HSCs: Hepatic stellate cells
α-KGDH: α-Ketoglutarate dehydrogenase
LDL-C: Low-density lipoprotein cholesterol
MDA: Malondialdehyde
MMP: Mitochondrial membrane potential
MTT: 3-(4,5-Dimethylthiazol-2-yl)-2,5-diphenyl tetrazolium bromide
NAM: Nicotinamide
NAD^+^: Nicotinamide adenosine dinucleotide
MASH: Metabolic dysfunction-associated steatohepatitis
Nrf2: Nuclear factor-erythroid 2 related factor 2
OXPHOS: Oxidative phosphorylation
PA: Palmitate
PBS: Phosphate buffer solution
PGC-1: Peroxisome proliferator-activated receptor γ coactivation factor 1
ROS: Reactive oxygen species
NADH: Reduced nicotinamide adenosine dinucleotide
SIRT1: Silent information regulator 1
α-SMA: α-Smooth muscle actin
SOD: Superoxide dismutase
SDH: Succinate dehydrogenase
SPF: Specific pathogen free
T-AOC: Total antioxidant capacity
TC: Total cholesterol
TEM: Transmission electron microscopy
TCA cycle: Tricarboxylic acid cycle
TG: Triglycerides
TGF-β: Transforming growth factor-β
