# Supplementary material for: Healthy mitochondria attenuate metabolic dysfunction-associated steatohepatitis by restoring cell metabolism
Source: Mol Biomed. 2025 Oct 11;6:80. doi: 10.1186/s43556-025-00328-w (PMC12515195; doi:10.1186/s43556-025-00328-w)
Supplement: Supplementary file 1 — Supplementary Material 1. [file 43556_2025_328_MOESM1_ESM.docx]

**Supplementary materials**

**Healthy mitochondria attenuate metabolic dysfunction-associated steatohepatitis by restoring cell metabolism**

Peiyu Zhou^#^, Jingli Li^#^, Yafang Xie^#^, Xiaorong Li, Zhihong Cui, Ailing Fu*

College of Pharmaceutical Sciences, Southwest University, Chongqing, China.

# The authors contributed equally to this paper.

* Corresponding author: Ailing Fu

E-mail: [fal@swu.edu.cn](mailto:pharmcy730808@163.com)

**1. Materials and Methods**

***Measurement of mitochondrial swelling***

The mitochondrial concentration was adjusted to achieve an absorbance of 0.8 - 0.9 at 540 nm. Then 1 mM CaCl_2_ (final concentration 200 μM) was added to the mitochondrial solution (800 μL). Subsequently, the absorbance was measured continuously for 30 min at 540 nm using an ultraviolet-visible spectrophotometer (ThermoFisher Scientific Inc., USA). The absorbance was recorded every 2 min. ***Distribution of exogenous mitochondria in vivo***

The isolated mitochondria were labeled with Mitotracker Red CMXRos (Beyotime, China; Catalog. C1035) according to the operation procedure. Then the mitochondria (0.2 mL) were slowly injected into mice at a dose of 0.2 mg/kg body weight *via* the tail vein. Two hours later, the mice were euthanized, and the liver, lung, kidney, and heart were dissected. The tissues were fixed with a 4% paraformaldehyde solution and then dehydrated with 10%, 20%, and 30% sucrose sequentially. Frozen sections (30 μm) were cut with a cryosectioning machine (Leica, Germany), and the fluorescence of the sections was observed under a fluorescence microscope (Chongqing Optec Instrument Co., Ltd, China).

***Dissolution of PA***

PA was dissolved in 0.1 mol/L NaOH solution in a 70°C water bath to prepare 100 mmol/L PA storage solution. Then the PA solution was mixed with the BSA solution at a volume ratio of 1:19 to form a 5 mmol/L PA/50 g/L BSA solution. The PA/BSA solution was placed in a 55°C water bath for 10 min and cooled to room temperature. The solution was diluted in cell culture medium to the final concentration before use. ***Western blot***

Total protein was extracted with RIPA lysis buffer, and the protein concentration was subsequently measured with a BCA assay kit (Sangon Biotech Co., Ltd., Shanghai, China). For sodium dodecyl sulfate-polyacrylamide gel electrophoresis (SDS-PAGE), 5% concentrated gels and 8% separated gels were prepared separately, and a 30 μg protein sample was subjected to electrophoretic separation. The protein sample was subsequently transferred to a polyvinylidene fluoride membrane with electrophoretic transfer. After the membrane was immersed in skim milk for 1 h, the primary antibody solution, PGC-1α (1:5000), was incubated with the membrane overnight. Beta-actin (1:1000) was used as a loading control. The membrane was washed with TBST buffer (1 M Tris-HCl, 137 mM NaCl, 2.7 mM KCl, 0.1% Tween-20; pH 7.5) and then incubated with horseradish peroxidase-linked secondary antibody buffer (1:10000) at 37°C for 1.5 h. Following washing with TBST buffer, the protein band on the membrane was developed with enhanced chemiluminescence.

***Catalog numbers of primary antibodies***

| **Name of primary antibody** | **Company** | **Catalog number** |
| --- | --- | --- |
| β-actin | Servicebio | GB11001-100 |
| collagen I | Servicebio | GB11022-3-100 |
| CPT1A | Proteintech | 15184-1-AP |
| FAS | Servicebio | GB15546-50 |
| Nrf2 | Beyotime | AF7623 |
| PGC-1α | Proteintech | 66369-1-IG |
| α-SMA | Servicebio | GB111364-100 |

***Catalog numbers of secondary antibody***

| **Name of *secondary* antibody** | **Company** | **Catalog number** |
| --- | --- | --- |
| Alexa Fluor 594 labeled goat anti-mouse IgG | Servicebio | GB28303 |
| Cy3-labeled goat anti-rabbit IgG | Servicebio | GB21303 |
| Fitc-labeled goat anti-rabbit IgG | Servicebio | GB22303 |
| HRP conjugated goat anti-mouse IgG | Servicebio | GB23301 |

***Mouse Diet***

Normal diet (Jiangsu Synergy Pharmaceutical Bioengineering Co.; Catalog number: XTI01WC-009)

High-fat diet: (Jiangsu Synergy Pharmaceutical Bioengineering Co.; Catalog number: XTMRCD60)

**2. Result**

**
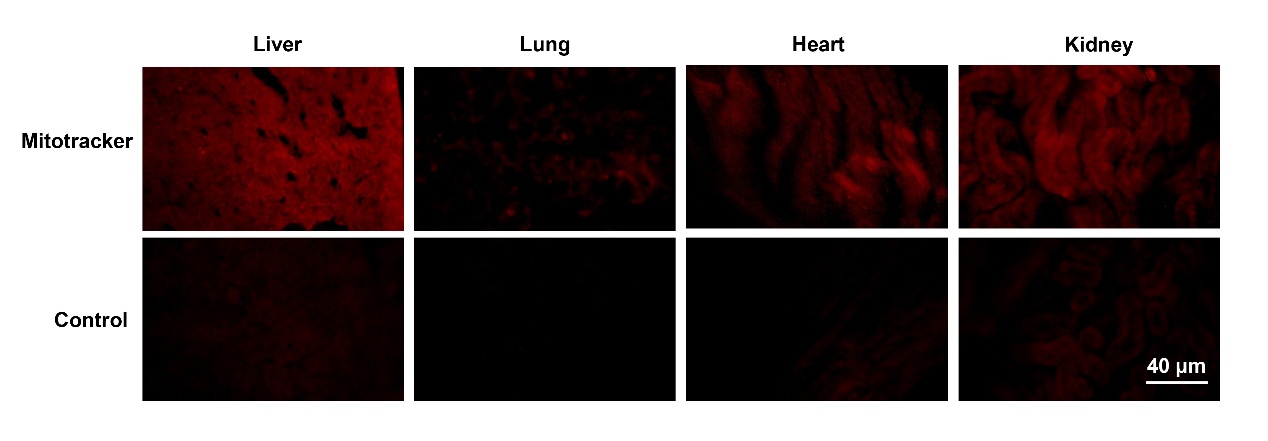
**

Figure S1. Distribution of the Mitotracker-labeled mitochondria after intravenous administration into mice.

**3. Western blot**

**
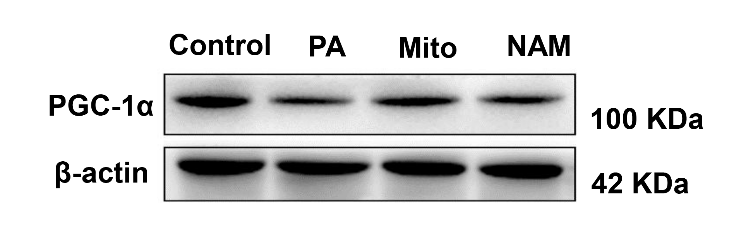
**

Figure S2. Detection of PGC-1α level by WB.

**
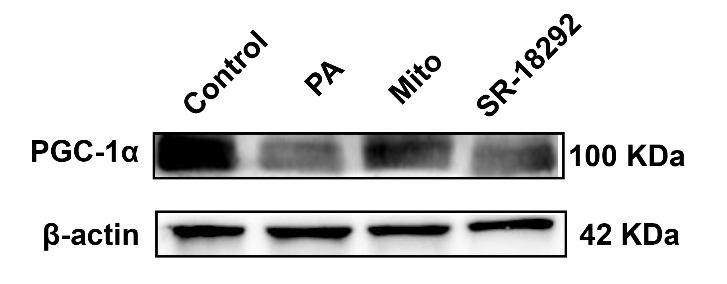
**

Figure S3. Detection of PGC-1α level by WB.


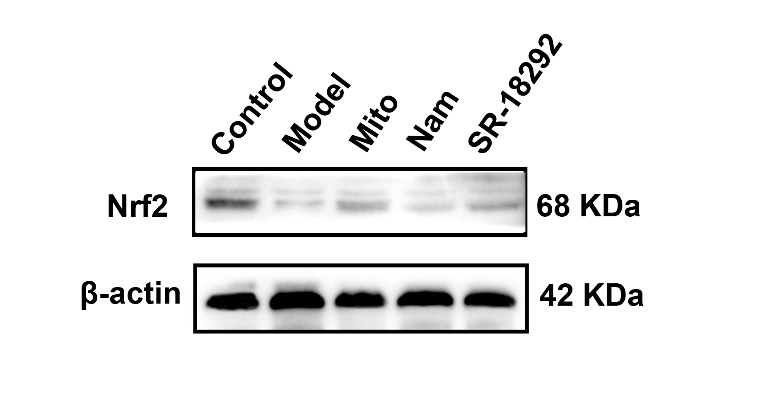


Figure S4. Detection of Nrf2 level by WB.


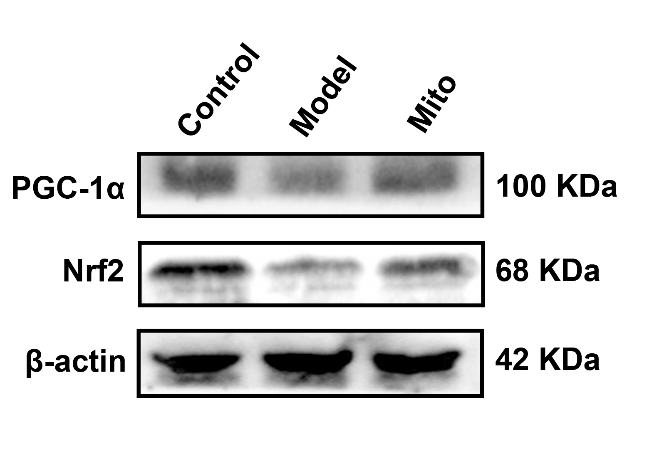


Figure S5. Detection of Nrf2 and PGC-1α levels by WB.
